# Prognostic markers of ferroptosis-related long non-coding RNA in lung adenocarcinomas

**DOI:** 10.3389/fgene.2023.1118273

**Published:** 2023-02-27

**Authors:** Kaimin Mao, Ri Tang, Yali Wu, Zhiyun Zhang, Yuan Gao, Huijing Huang

**Affiliations:** ^1^ Department of Critical Care Medicine, Renji Hospital, School of Medicine, Shanghai Jiaotong University, Shanghai, China; ^2^ Department of Respiratory and Critical Care Medicine, NHC Key Laboratory of Pulmonary Diseases, Union Hospital, Tongji Medical College, Huazhong University of Science and Technology, Wuhan, China; ^3^ Department of Rheumatology, Zhongshan Hospital, Fudan University, Shanghai, China

**Keywords:** ferroptosis, lncRNA, lung adenocarcinoma, risk scores model, immunotherapy

## Abstract

Ferroptosis is a recently established type of iron-dependent programmed cell death. Growing studies have focused on the function of ferroptosis in cancers, including lung adenocarcinoma (LUAD). However, the factors involved in the regulation of ferroptosis-related genes are not fully understood. In this study, we collected data from lung adenocarcinoma datasets of the Cancer Genome Atlas (TCGA-LUAD). The expression profiles of 60 ferroptosis-related genes were screened, and two differentially expressed ferroptosis subtypes were identified. We found the two ferroptosis subtypes can predict clinical outcomes and therapeutic responses in LUAD patients. Furthermore, key long non-coding RNAs (lncRNAs) were screened by single factor Cox and least absolute shrinkage and selection operator (LASSO) based on which co-expressed with the 60 ferroptosis-related genes. We then established a risk score model which included 13 LUAD ferroptosis-related lncRNAs with a multi-factor Cox regression. The risk score model showed a good performance in evaluating the outcome of LUAD. What’s more, we divided TCGA-LUAD tumor samples into two groups with high- and low-risk scores and further explored the differences in clinical characteristics, tumor mutation burden, and tumor immune cell infiltration among different LUAD tumor risk score groups and evaluate the predictive ability of risk score for immunotherapy benefit. Our findings provide good support for immunotherapy in LUAD in the future.

## Introduction

Lung cancer is one of the most common malignant tumors and the leading cause of cancer-related deaths worldwide. Despite the continuous emergence of new treatments, the prognosis of lung cancer is still very poor (Siegel et al., 2020). Non-small-cell lung cancer (NSCLC) is the main histologic subtype of lung cancer, it can be classified as lung adenocarcinoma (LUAD), lung squamous cell carcinoma (LUSC), and large-cell carcinoma, of which LUAD is the most common subtype (Relli et al., 2019). It is important to identify effective biomarkers for the prognosis of LUAD because, even though there are a variety of treatment plans for this cancer, the average 5-year survival rate is only about 15% (Spella and Stathopoulos, 2021).

Ferroptosis is a new type of iron-dependent programmed cell death that differs from apoptosis, necrosis, and autophagy. It induces cell injury or death *via* the iron-dependent lipid peroxidation process (Latunde-Dada, 2017; Xu et al., 2023). Ferroptosis is characterized by increased mitochondrial membrane density and cell volume contraction, which is different from other morphological, biochemical, and genetically regulated cell deaths (Hassannia et al., 2019; Li et al., 2020). Studies have shown that ferroptosis inhibits tumor growth, kills tumor cells, and prevents tumor migration (Mou et al., 2019). Accumulating evidence has suggested that ferroptosis is associated with several biological processes in LUAD. For example, CAMP-responsive element binding protein 1 (CREB) can directly bind to the promoter region of glutathione peroxidase 4 (GPX4) to promote its expression, thereby inhibiting potential ferroptosis and promoting the growth of LUAD (Wang Z. et al., 2021). Besides, the novel 15-gene signature of ferroptosis provides a basis for an accurate prediction of the prognosis of LUAD, allowing for the development of new therapies and personalized outcome prediction in this population (Zhang A. et al., 2021). Therefore, it is necessary to find new treatment strategies to improve the prognosis of LUAD by regulating ferroptosis.

Recent advances in sequencing technologies have shown that 90% of RNAs do not encode proteins, which are called non-coding RNA (ncRNA) (Matsui and Corey, 2017). Long ncRNA (LncRNA) is a type of ncRNA. It has a length of more than 200 nucleotides and is mainly involved in regulating gene promoters and enhancers as well as RNA splicing (Ali and Grote, 2020). Several studies have indicated that RNA plays an important role in the development of cancer, its metastatic and genital development, and so it is now an important candidate for cancer treatment (Li et al., 2016; Liu S. J. et al., 2021). What’s more, lncRNAs are increasingly recognized as crucial mediators in the regulation of ferroptosis (Gibb et al., 2011). For example, Chao Mao et al. demonstrated that the cytosolic lncRNA P53RRA promotes ferroptosis and apoptosis in lung cancer *via* nuclear sequestration of p53 (Jiang et al., 2015). In addition, it was demonstrated that lncRNA LINC00336, which is associated with ferroptosis, is highly expressed in lung cancer, and acts as a competitive endogenous RNA to function as an oncogene (Wang et al., 2020). However, the full role of ferroptosis-related lncRNAs in LUAD is still not completely understood. For new therapeutic strategies for patients with LUAD, ferroptosis-related lncRNAs must be identified to predict their outcome.

Anti-tumor immune response has long been a fundamental strategy in cancer immunotherapy (Liang et al., 2021). While ferroptosis plays a key role in tumor immunity. Therefore, it is important to explore biomarkers associated with tumor immunity and ferroptosis for immunotherapy of lung cancer. In this study, a ferroptosis-related lncRNA signature associated with LUAD prognosis is being explored based on the LUAD dataset of TCGA. To predict the survival of LUAD patients, a ferroptosis-related lncRNA risk score model was established by univariate and multivariate Cox regression analysis. In addition, the acting mechanism of ferroptosis-related lncRNAs in tumor progression was further mined by functional analysis and immune infiltration analysis to provide new insights into the prognosis and immunotherapy of LUAD. Our study provides insights into the mechanisms underlying ferroptosis in the treatment of LUAD, which may improve individualized therapy and the assessment of prognosis for LUAD.

## Materials and methods

### Acquisition of gene expression and clinical data

**FIGURE 1:**
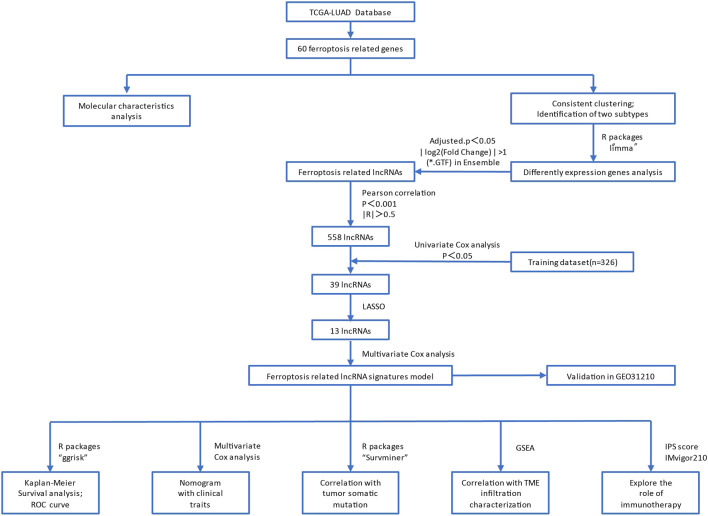
The flow chart of this study.

The process flow of this study is shown in Figure 1. Briefly, the LUAD expression profiles and clinical follow-up information were downloaded from the TCGA database (https://portal.gdc.cancer.gov/). The RNA-Seq data of TCGA-LUAD was processed in the following steps. Samples without clinical follow-up information and survival time were removed. We also excluded patients who survived less than 30 days and with no survival status. We converted probes to Gene Symbol, with one probe corresponding to multiple genes. Besides, we used the median value for the expression of multiple Gene Symbols. Finally, 489 tumor samples were included from the pre-processed TCGA-LUAD, as shown in Supplementary Table S1.

### Consensus clustering of tumor ferroptosis-related gene expression

Ferroptosis is a new type of programmed cell death that differs from apoptosis, necrosis, and autophagy. As a result of divalent iron or ester oxygenase action, it causes unsaturated fatty acids highly expressed on the cell membrane to undergo lipid peroxidation, thus leading to cell death. Aside from this, it also acts as an antioxidant system (glutathione), which reduces the GPX4 enzyme. To ensure the stability of the classification, we used the ConsensuClusterPlus package in R and the Pam method based on Euclid and Ward linkages.

### Differentially expressed genes among tumor ferroptosis subtypes (Fer_DEGs)

Two groups of samples of Fer-1 and Fer-2 were acquired based on the expression of tumor ferroptosis-related genes and consistent clustering results. The screening threshold was set as adjusted. *P* < 0.05 and | log2 (Fold Change) | > 1. A differentially expressed gene was analyzed between two subtypes using the “limma” package in R software. In addition, the Ensemble display was used to extract lncRNAs from differentially expressed genes.

### Gene ontology and kyoto encyclopedia of genes and genomes pathway enrichment analyses

The co-expression genes of differential ferroptosis-related genes between high- and low-risk LUAD patients were chosen to perform Gene Ontology (GO) and Kyoto Encyclopedia of Genes and Genomes (KEGG) analyses, which was conducted by using the clusterProfifiler package. Enrichment significance thresholds were set at *p* < 0.05 and false discovery rate (FDR) < 0.05 (Guo X. H. et al., 2021; Cao et al., 2021b). GO analysis was used to map all DEGs to GO terms in the GO database (http://www.geneontology.org/) to analyze the main functions of the DEGs. The KEGG pathway database (http://www.geneontology.org/) is a synthetic database, which was used to analyze the biochemical pathways of the DEGs of interest (Zhong H. et al., 2021).

### Construction of ferroptosis-related lncRNA risk score model

To calculate the risk score for LUAD, we constructed a model based on the lncRNAs associated with ferroptosis subtypes. To reduce noise or redundant genes, a univariate Cox algorithm was applied to narrow the lncRNA set associated with immune cell infiltration subtypes. The best prognostic signature was identified by using the Lasso method [Least absolute shrinkage and selection operator, Tibshirani (1996)] A multi-factor Cox regression analysis contributed to the development of a risk score model for tumor immune cell infiltration. The formula was as follows:

$$Risk\_scores=\sum Coef\left(i\right)*\mathrm{Exp}\left(i\right)$$

### Gene set enrichment analysis (GSEA)

GSEA was published in 2005 based on gene set enrichment analysis. Genome-wide expression profiles can be interpreted using this knowledge-based approach. Using MSigDB (gene matrix transposition file format *.gmt) we selected one or more functional gene sets to analyze gene expression data (Guo Y. et al., 2021). We then sorted the gene expression data by correlation degree of phenotype (also known as a change in expression amount). To evaluate the influence of synergistic changes in genes on phenotypic changes, we sorted by phenotypic relevance the genes enriched in the upper and lower parts of the gene list.

### Independent prognostic factors analysis of risk score and construction of a nomogram prediction model

After the extraction of clinical information (including age, gender, smoking, and TNM stage) of LUAD patients in the TCGA, univariate and multivariate prognostic analyses were used to demonstrate whether the risk score could be an independent prognostic factor. Based on the multivariate Cox regression analysis for risk score and other clinicopathological factors by the rms R package, a clinically adaptable nomogram prediction model was established to predict the survival probability of 489 LUAD individuals in 1-, 3-, and 5- years from the TCGA group. Then, the calibration analysis and time-dependent ROC curve were used to evaluate the prognostic value of the nomogram for LUAD patients (Sun et al., 2022).

### Analysis of the tumor mutation burden in the high- and low-tumor risk score groups

Tumor mutational burden (TMB) is broadly defined as the number of somatic mutations per megabase of interrogated genomic sequence (Bravaccini et al., 2021). To inquire about the association between the TMB and tumor risk score, we compared the tumor mutation status between the low- and high-risk score groups. The somatic mutation file *.maf of TCGA-LUAD was downloaded from the GDC Data Portal (https://portal.gdc.cancer.gov) to calculate the TMB values. Significantly mutated genes (*p* < 0.05) between the low- and high-risk groups and the interaction effect of gene mutations were analyzed by maftools; only genes mutating more than 50 times in at least one group will be considered. The statistical significance test for the proportion of mutation was evaluated by Pearson correlation coefficient, student *t* test, Chi-square test, and survival analysis.

### Relationship between tumor risk score and tumor microenvironment

Based on the LM22 signature and 1,000 permutations, the mutations of 22 different immune cells in TCGA-LUAD (B.cells.naive, B.cells.memory, Plasma.cells, T.cells.CD8, T.cells.CD4.naive, T.cells.CD4.memory.resting, T.cells.CD4.memory.activated, T.cells.follicular.helper, T.cells.regulatory.Tregs, T.cells.gamma.delta, NK.cells.resting, NK.cells.activated, Monocytes, Macrophages.M0, Macrophages.M1, Macrophages.M2, Dendritic.cells.resting, Dendritic.cells.activated, Mast.cells.resting, Mast.cells.activated, Eosinophils, Neutrophils) infiltration levels were quantified by using the CIBERSORT package in R. Besides, differences in the degree of immune cell infiltration between high- and low-risk groups were compared.

### Correlation analyses between tumor risk score and immunotherapy response

The correlation between tumor risk score and immunotherapy response can evaluate the effect of the tumor risk score in predicting the benefit of immunotherapy in treating LUAD patients. In this study, we compared the immunotherapy response between the high- and low-risk groups based on expression profile data and clinical information in the IMvigor210 cohort (http://research-pub.gene.com/IMvigor210CoreBiologies/).

### Reverse transcription-quantitative PCR (RT-qPCR)

Five paired LUAD tissues and corresponding adjacent non-tumorous tissues were obtained from patients who underwent radical resection of lung cancer in Renji hospital, Total RNA was extracted with TRIzol™ Reagent (Invitrogen). Reverse transcription of RNA was performed using PrimeScript™ RT Master Mix (Takara). In this study, Takara’s TB Green™ Premix EX Taq™ II was used to perform the qPCR. GAPDH was used as an internal control (Cao et al., 2021a; Fei et al., 2021). The primer sequence of the tested genes is shown in Supplementary Table S6. The relative lncRNA expression level was quantified using the 2^−ΔΔCt^ method.

### Statistical analysis and hypothesis testing

All statistical comparisons involved in this study, as well as hypothesis testing of the significance of differences between groups, were based on the statistical analysis method in R 3.6.

## Results

### Molecular characteristics of ferroptosis-related genes in LUAD

**FIGURE 2:**
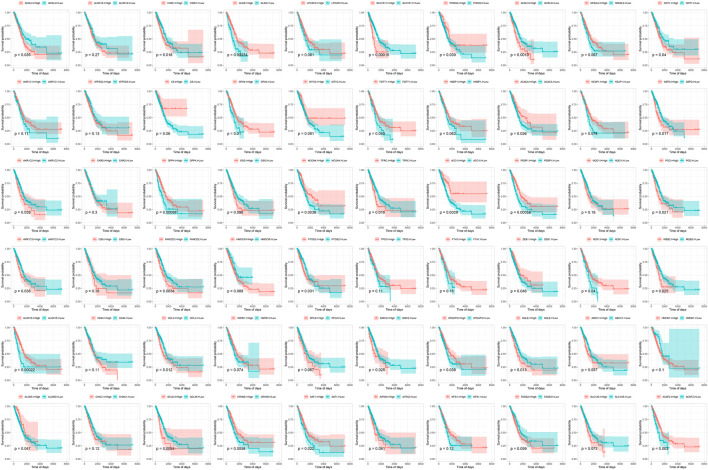
Survival curve of 60 ferroptosis-related genes and overall survival in the TCGA-LUAD data set.

The flow chart of this study was shown in Figure 1. Based on the expression values of 60 ferroptosis-related genes in each sample of the TCGA-LUAD dataset, the genes were divided into a high-expression group and a low-expression group according to the optimal density algorithm. The high expressions of GLS2, PHKG2, ACACA, GPX4, DPP4, NCOA4, ACO1, PEBP1, NOX1, ZEB1, ALOX15, ALOX5, CRYAB, SAT1, and ACSF2 are significantly associated with better OS prognosis. While the low expressions of GCLM, GCLC, EMC2, SQLE, IREB2, FANCD2, AKR1C3, AKR1C2, TFRC, PGD, G6PD, ACSL4, CISD1, SLC7A11, ACSL3, and GOT1 have great significance with better OS prognosis (Figure 2).

Subsequently, the statistics of gene mutations in the TCGA-LUAD showed that 88.95% of tumor samples had gene mutations, including 47% of TP53 mutations, 41% of TTN mutations, 40% of MUC16 mutations, and 34% of RYR2 mutations (Supplementary Figure S1).

Furthermore, we conducted a hypothesis test on whether TP53 and TTN affect the expression of 60 ferroptosis-related genes. We found that the mutation of the TP53 gene was significantly associated with the high expression of CBS, GCLM, FANCD2, GSS, HSPB1, MT1G, TFRC, SQLE, FADS2, and NFS1 genes, while it has a remarkable correlation with the low expression of PEBP1, TP53, FDFT1, SLC7A11, CRYAB, NCOA4, SAT1, GLS2, AKR1C1, and AKR1C3 (Supplementary Figure S2). Among the mutation groups with TTN, ATP5G3, CARS, CBS, GPX4, GCLM, GCLC, FANCD2, CS, CISD1, CHAC1, GSS, HSPB1, RPL8, ACO1, EMC2, TFRC, NFS1, ZEB1, SQLE, FADS2, IREB2, PGD, and SLC1A5 were significantly highly expressed, while ALOX5, CD44, CRYAB, and SAT1 showed a significantly low expression status (Supplementary Figure S3). At the same time, we observed that most of the expressions of 60 ferroptosis-related genes were mutually promoting, as shown in Supplementary Figure S4.

### Identification of ferroptosis subtypes and differentially expressed genes in LUAD

**FIGURE 3:**
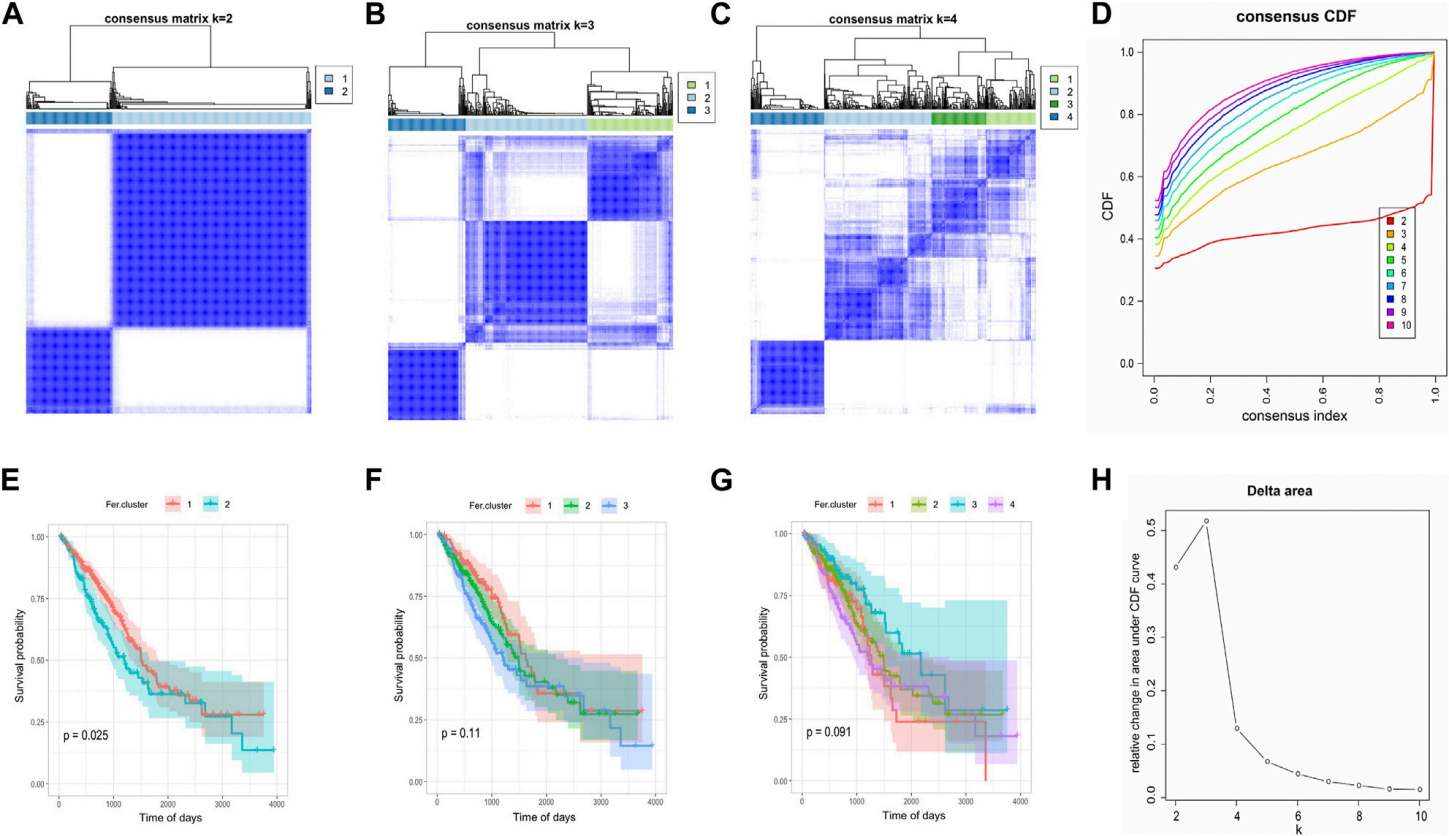
Consensus clustering of tumor ferroptosis-related genes expression profiles. **(A–C)** The clustering results when consensus matrix k = 2, k = 3, and k = 4. **(D)** Distribution of CDF curve of consensus clustering. **(E–G)** Survival curve when consensus matrix k = 2, k = 3, and k = 4, respectively. **(H)** Distribution of area under the CDF curve of consensus clustering.

Consensus clustering was performed based on the expression of 60 ferroptosis-related genes in the TCGA-LUAD, and we determined two independent ferroptosis subtypes with a significant difference in survival. Among the two ferroptosis subtypes, Fer-1 has a significantly better prognosis than Fer-2, with a median survival time of 898 days. While Fer-2 indicated a worse disease prognosis, with a median survival time of 685 days (Figure 3).

**FIGURE 4:**
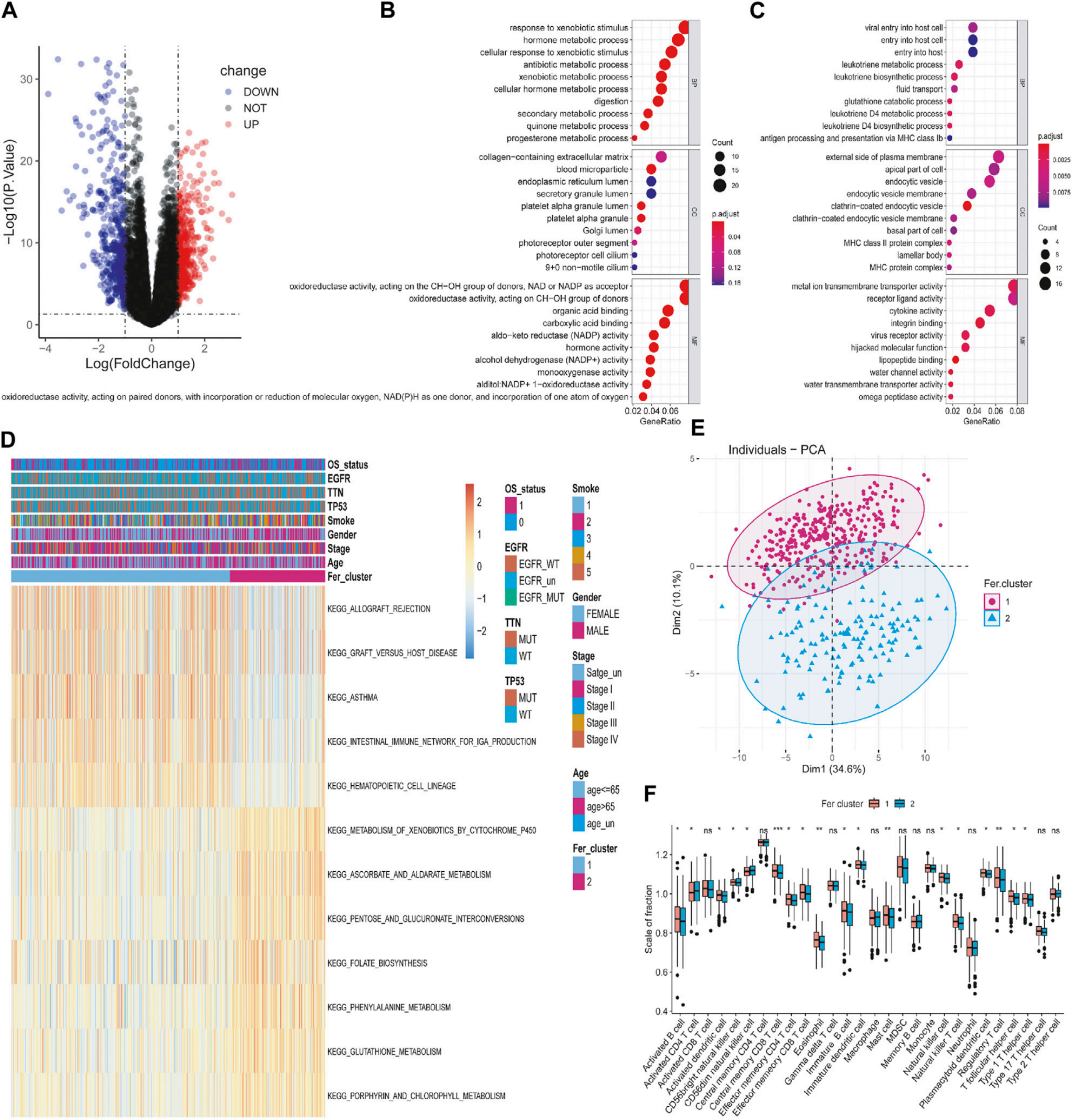
Identification and functional analysis of differentially expressed genes among different tumor ferroptosis subtypes. **(A)** Volcano map of differential expressed genes. **(B,C)** Bubble chart of GO enrichment analysis of upregulated and downregulated genes. **(D)** KEGG enrichment analysis of gene set. **(E)** PCA analysis of expression profile. **(F)** Tumor immune cell infiltration analysis of the gene dataset.

In order to reveal the potential biological characteristics of different ferroptosis states, we used the “limma” package of R software to analyze differentially expressed genes between the subtypes. 882 genes were identified with an adjusted *p* < 0.05 and | log2 (Fold Change) | >1 (Supplementary Table S2). Among them, 511 genes were highly expressed in Fer-1 subtypes, while 371 genes were upregulated in Fer-2 (Figure 4A). Subsequently, we performed the Gene Ontology (GO) functional enrichment analysis on highly expressed genes. The first 10 pathways enriched in the three functional categories (BP, CC, and MF) were displayed with bubble diagrams (Figures 4B, C). Most of the pathways in Fer-1 were correlated with biological processes such as response to xenobiotic stimulus, hormone metabolic process, and antibiotic metabolic process. While in Fer-2, most of the enrichments were related to viral entry into the host cell, leukotriene metabolic process, and fluid transport.

Then, we performed Kyoto Encyclopedia of Genes and Genomes (KEGG) pathway enrichment analysis on the DEGs, and the first 12 enriched pathways were determined. As shown in Figure 4D, they were allograft rejection, graft *versus* host disease, asthma, intestinal immune network for iga production, hematopoietic cell lineage, metabolism of xenobiotics by cytochrome p450, ascorbate and aldarate metabolism, pentose and glucuronate interconversions, folate biosynthesis, phenylalanine metabolism, glutathione metabolism, porphyrin metabolism, and porphyrin metabolism, porphyrin metabolism. To further explore the relationship between tumor ferroptosis subtypes and tumor immune cells, firstly, we used principal component analysis (PCA) algorithm to visualize the expression profiles related to ferroptosis subtypes. As shown in Figure 4E, it is found that the samples in the first dimension and the second dimension have a good aggregation form, which indicates that the classification method of ferroptosis subtypes is reasonable. Secondly, as shown in Figure 4F, by comparing the immune cells infiltrating the difference between ferroptosis subtypes, it was found that mast cells, immature B cells, eosinophil, activated B cells, activated dendritic cells, and immature dendritic cells were significantly infiltrated at a high level in Fer-1 compared with Fer-2. In summary, the expression profile of ferroptosis-related genes in LUAD is consistent with the prognosis profile, indicating that it was a viable method to classify ferroptosis subtypes.

### The construction of LUAD ferroptosis-related lncRNA risk score model

**FIGURE 5:**
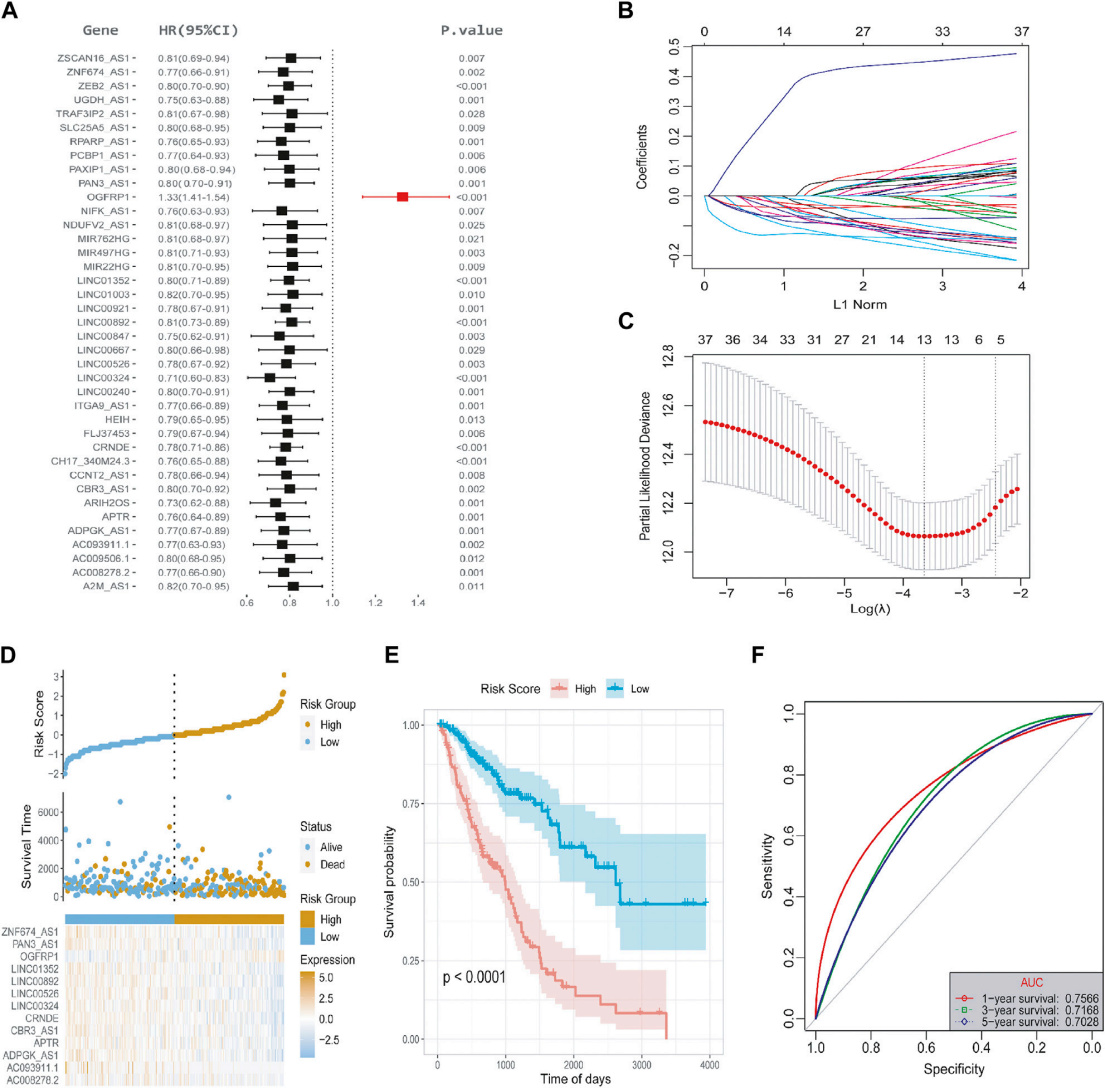
Screening of lncRNAs and the construction of risk score model. **(A)** Univariate Cox regression analysis was used to identify 558 candidate lncRNAs in the training set, and 39 lncRNAs were retained. The meaningful threshold was set as *p*-value < 0.05. **(B)** The changing trajectory of each independent variable. The horizontal axis represents the log value of the independent variable lambda, and the vertical axis represents the coefficient of the independent variable. **(C)** The confidence interval under each lambda. **(D)** The risk score distribution diagram; **(E)** Survival curve of LUAD patients with high- and low-risk scores. **(F)** ROC curve at 1-, 3-, and 5- years.

To explore the expression of ferroptosis-related lncRNAs and their role in the evaluation of OS of LUAD, we used the Pearson correlation coefficient to identify lncRNAs that co-expressed with ferroptosis-related genes (*P*-value <0.001 and |R| > 0.5). As a result, 558 lncRNAs were screened which have a significant co-expression relationship with at least one ferroptosis gene (Supplementary Table S3). In this study, we constructed a risk score model of tumor immune cell infiltration based on the ferroptosis-related lncRNAs. Firstly, according to an approximate 2:1 ratio, the TCGA-LUAD overall set (n = 489) was divided into a training set (n = 326) and a test set (n = 163). In the training set, we displayed univariate Cox analysis to analyze 558 candidate lncRNAs. As shown in Figure 5A, 39 lncRNAs were retained with a meaningful threshold of p.value < 0.05 (Supplementary Table S4). For the convenience of clinical application, 13 lncRNAs were identified by LASSO regression (Figures 5B, C). Multivariate Cox regression was used to construct the lncRNA risk score model based on the 13 lncRNAs, The final 13-lncRNA gene signature formula is as follows:

$$\begin{array}{l} Risk\;score=\left(-0.041\right)\times AC008278.2+\left(-0.098\right)\times AC093911.1+\left(-0.132\right)\times ADPGK-AS1+\left(-0.060\right)\\ \times APTR+\left(-0.074\right)\times CBR3-AS1+\left(-0.122\right)\times CRNDE+\left(-0.072\right)\times LINC00324+\left(-0.088\right)\times LINC00526+\left(-0.041\right)\\ \times LINC00892+\left(-0.109\right)\times LINC01352+\left(0.454\right)\times OGFRP1+\left(-0.021\right)\times PAN3-AS1+\left(-0.088\right)\times ZNF674-AS1 \end{array}$$

An R package called “ggrisk” was used to evaluate the power of the risk score model in predicting OS. Based on the optimal density gradient algorithm, patients were divided into high-risk and low-risk groups. The high-risk group had a higher mortality rate, as shown in Figure 5D. Kaplan-Meier survival analysis showed that the high-risk group has a significantly lower OS than the low-risk group (Figure 5E). The receiver operating characteristic curve (ROC) curves in Figure 5F indicated that the area under the curve (AUC) at TCGA-LUAD data sets was 0.7566, 0.7128, 0.7028 at 1-, 3-, and 5- years, respectively, indicating that the risk score is capable of predicting overall survival.

**FIGURE 6:**
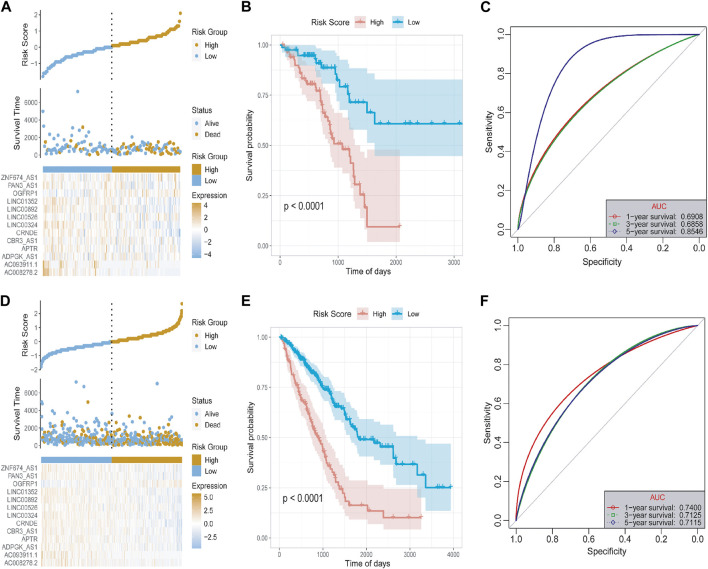
Test set and overall set to verify the risk model. **(A)** The distribution diagram of the risk score of the test set. **(B)** The survival curve of the test set. **(C)** The 1-, 3-, and 5- year’s ROC curves of the test set. **(D)** Distribution chart of the risk score of the overall set. **(E)** Survival curve of the overall set. **(F)** The 1-, 3-, and 5- year’s ROC curve of the overall set.

Subsequently, we used the test set and the overall set of TCGA-LUAD to access the predictive ability of risk score on OS. Based on the optimal density gradient algorithm, we assigned the patients to high-risk groups and low-risk groups. As shown in Figures 6A, D, the proportion of death samples in the high-risk group was relatively high. As Kaplan-Meier analyzed, the high-risk group has a significantly lower OS than the low-risk group (Figures 6B, E), suggesting that in the test set, the risk score model has a good predictive value. Its 1-, 3-, and 5- year AUC reached 0.6908, 0.6858, and 0.8546, respectively (Figure 6C). Similarly, in the overall dataset of TCGA-LUAD, the risk score model also has a good predictive value, with the 1-, 3-, and 5- year’s AUC of 0.7400, 0.7125, and 0.7115, respectively (Figure 6F).

**FIGURE 7:**
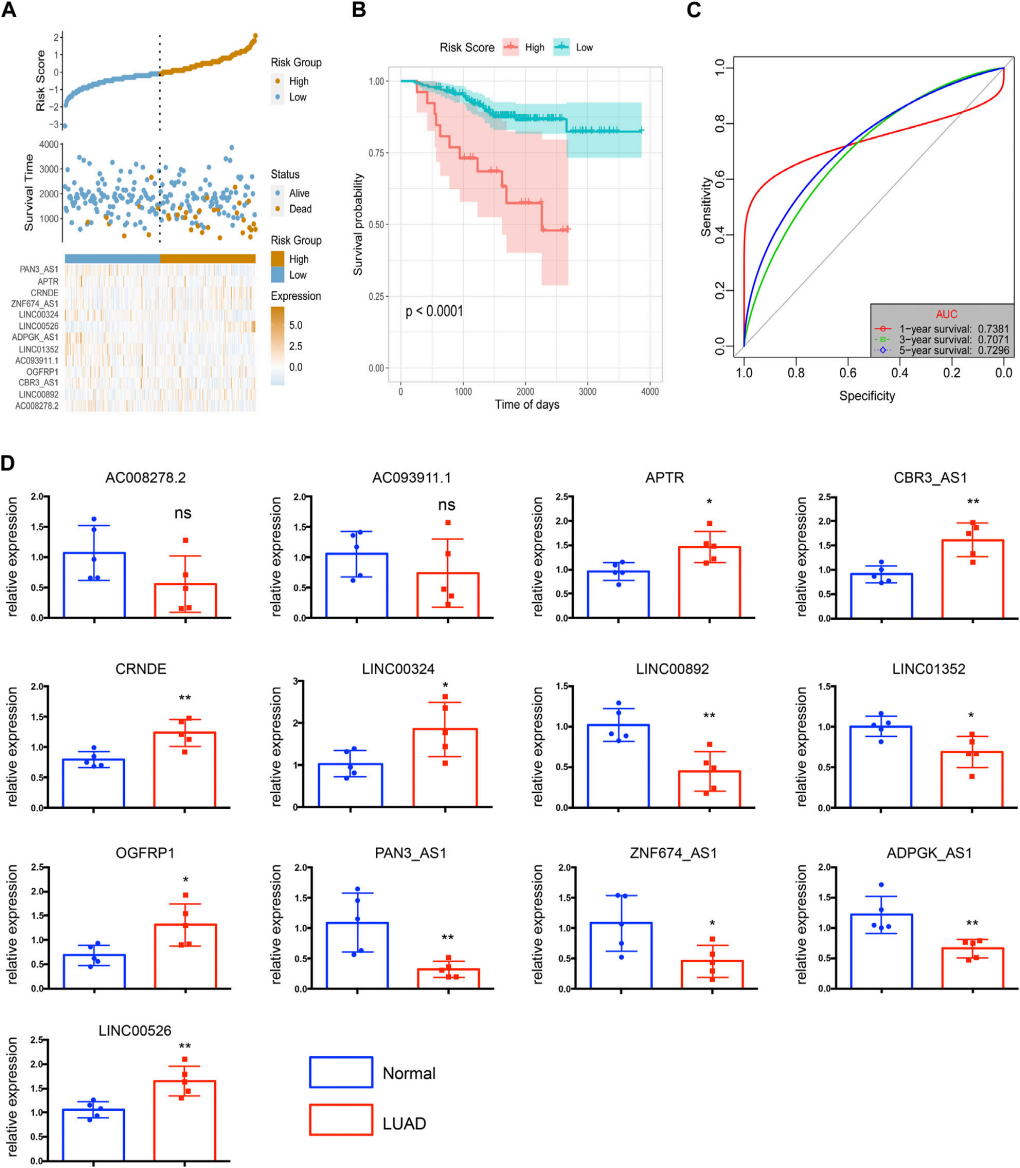
The risk score model was validated by the external dataset GSE31210. **(A)** The distribution of risk scores. **(B)** The survival probability was higher in the high-risk group compared with the low-risk group. **(C)** The 1-, 3-, and 5-year ROC curves of the external dataset. **(D)** Relative expressions of 13 key lncRNAs in LUAD tissues (LUAD) and corresponding adjacent non-tumorous tissues (normal). *N* = 5 in each group. *p < 0.05, **p < 0.01, ***p < 0.001, ns = no significance.

To evaluate the robustness of the risk score model in predicting OS of LUAD, the risk score model was validated by the external dataset GSE31210. By using the ggrisk software package in R, the samples were divided into high-risk and low-risk groups based on the optimal density gradient algorithm. We found that the proportion of death in the high-risk group was higher compared with the low-risk group (Figure 7A). In addition, Kaplan-Meier analysis showed that the OS of patients in the high-risk group was significantly lower than that in the low-risk group (Figure 7B). Therefore, the risk score model was also robust in predicting OS in the GSE31210 dataset (Figure 7C). The 1-, 3-, and 5- year’s AUC was 0.7381, 0.7071, and 0.7296, respectively.

To better estimated the above bioinformatics results obtained from the public databases, we detected the levels of 13 key lncRNAs by using 5 paired LUAD tissues and corresponding adjacent non-tumorous tissues. The quantitative RT-qPCR array in LUAD tissues shows enhanced expression of upregulated lncRNAs including APTR, CRNDE, LINC00324, OGFRP1, and LINC00526, as shown in Figure 7D. In contrast, LINC00892, LINC01352, PAN3-AS1, ZNF674-AS1, and ADPGK-AS1 have significantly diminished in non-tumorous tissues. Because of limited samples, we did not observe a significant difference in the expression of AC008278.2 and AC093911.1 in LUAD and non-tumorous tissues.

### The relationship between risk score and clinical characteristics

**FIGURE 8:**
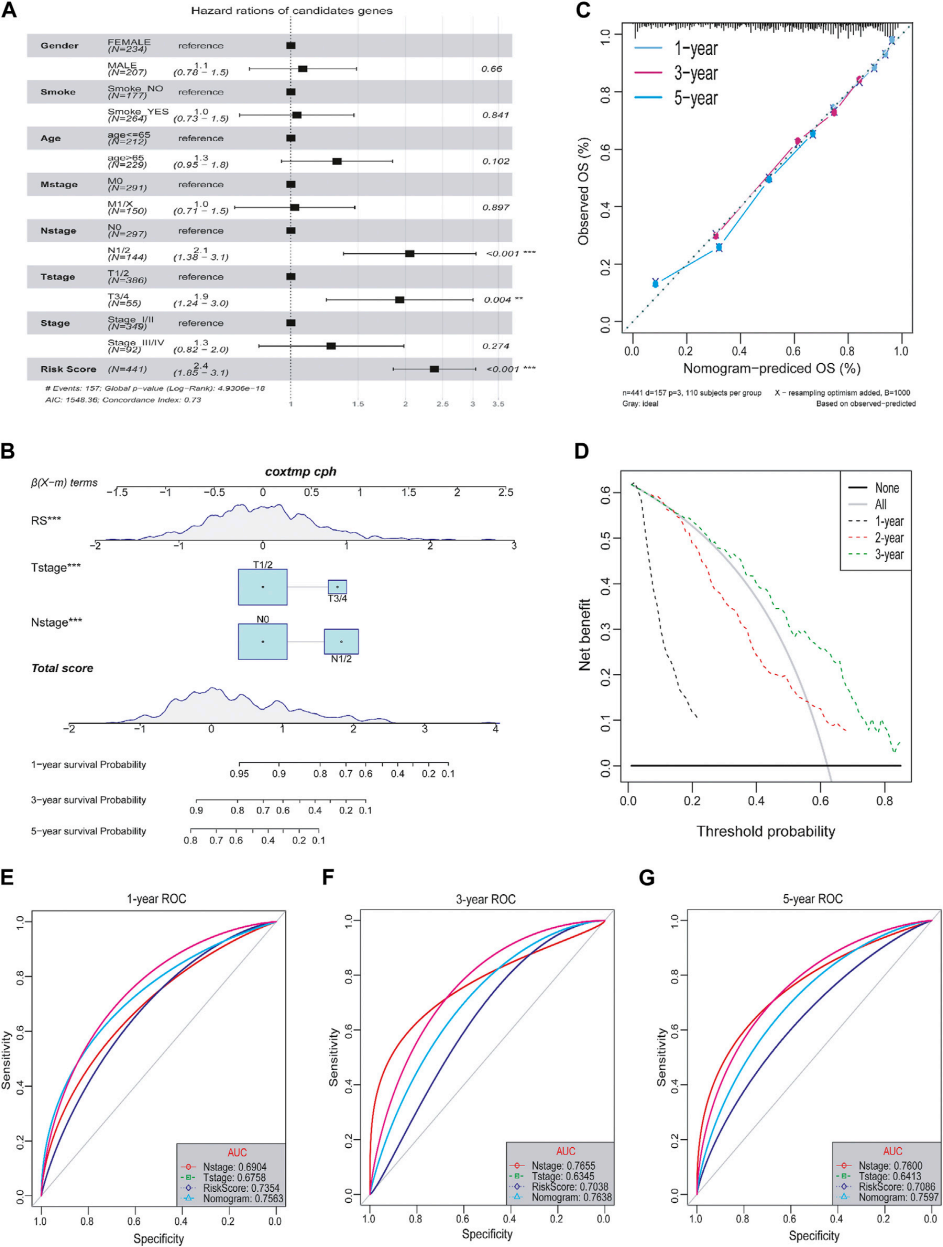
Relationship between tumor risk score and clinical characteristics. **(A)** Multivariate cox analysis of clinical characteristics and risk score. **(B)** Nomograms of clinical characteristics and risk score. **(C)** Calibration charts of nomograms in 1-, 3-, and 5-year. **(D)** DCA distribution map of nomograms in 1-, 3-, and 5-year. **(E–G)** ROC curves in 1-, 3-, and 5-year.

It is necessary to clarify the relationship between tumor risk score and clinical characteristics, including age, smoke, and tumor grade. Firstly, multivariate Cox analysis determined that the lncRNA risk score was independent of other prognostic factors, such as age, gender, smoke and tumor stage, M-sage, N-stage, and T-stage (Figure 8A). Next, for the convenience of clinical evaluation, we construct a nomogram by using the risk score, T-stage, and N-stage (Figure 8B). The calibration curves of the nomogram 1-, 3-, and 5- years showed good stability. Notably, the ROC curve suggested that the predictive ability of the nomogram was higher than other factors (Figures 8C, D), with the AUC values reaching a high level above 0.75 (Figures 8E–G). Therefore, the lncRNA-based risk score was a relatively independent prognostic indicator in LUAD.

### The relationship between lncRNA risk score and tumor mutation burden

**FIGURE 9:**
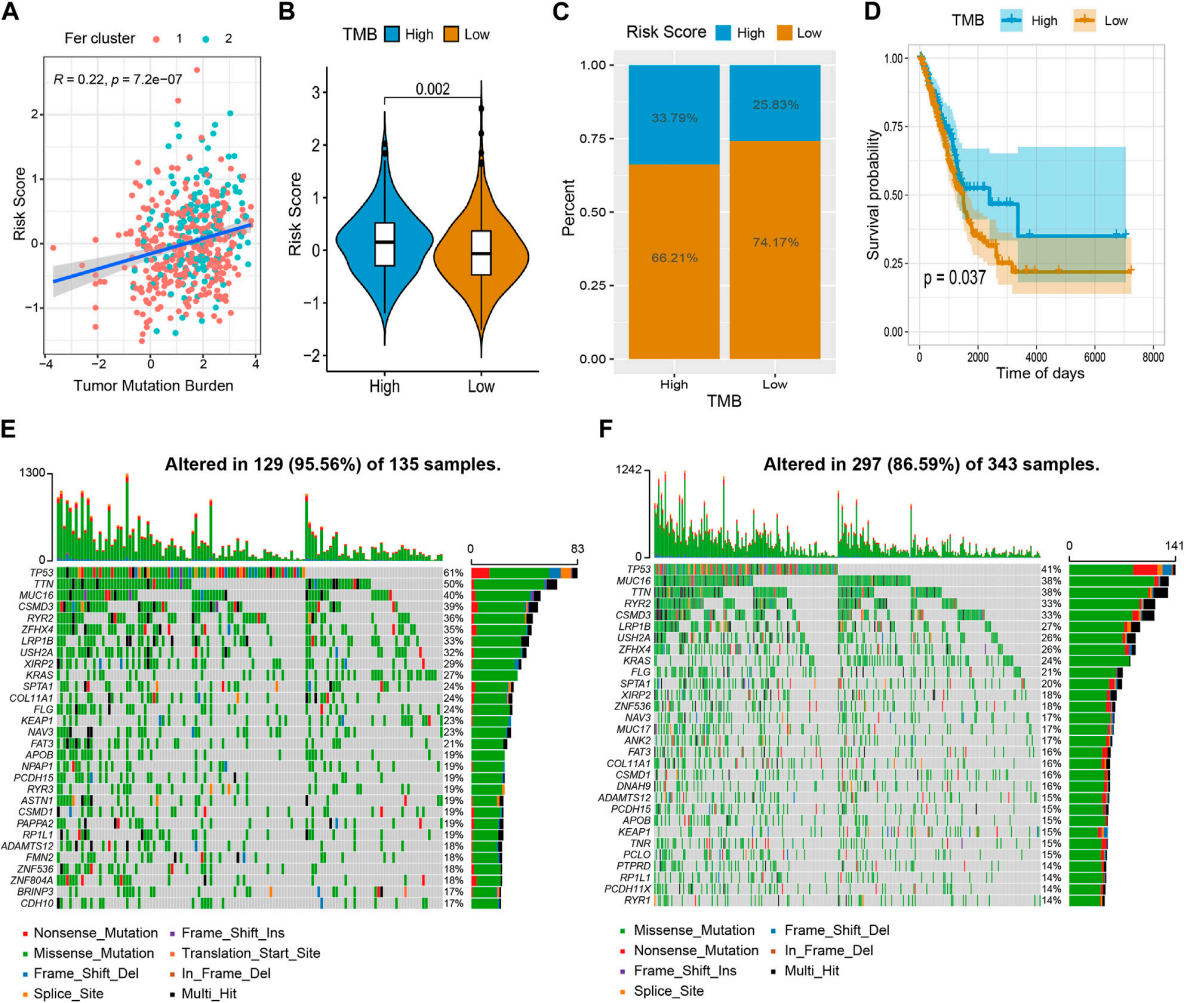
The relationship between tumor risk score and tumor mutation burden. **(A)** The risk score was positively correlated to TMB. **(B,C)** Violin chart and proportional distribution bar chart showed that TMB was higher in the high-risk score group than in the low-risk score group. **(D)** The survival curve showed patients in the high TMB have a better survival probability than the low TMB group. **(E)** High-risk group gene mutation waterfall chart. **(F)** Low-risk group gene mutation waterfall chart.

Growing evidence suggests that tumor mutation burden (TMB) may determine the individual response to cancer immunotherapy (Bravaccini et al., 2021). It is important to explore the relationship between TMB and risk score to clarify the genetic characteristics of each ferroptosis subgroup. Correlation analysis (Figure 9A) showed that risk score was positively associated with TMB (R = 0.22, *p* = 7.2 × 10^−7^). By comparing the TMB of patients in subgroups (Figures 9B, C), we found that TMB in the high-risk score group was higher than in the low-risk score group. Furtherly, we used the Survminer package in R to calculate the optimal density gradient threshold associated with TMB and survival, and divided tumor samples in TCGA-LUAD into two groups with high- and low- TMB scores. As a result, we found a remarkable difference in survival between the two groups, as shown in Figure 9D.

In addition, we quantified the distribution of somatic variation in LUAD driver genes between low-risk and high-risk score groups, meanwhile, the top 30 driver genes with the highest mutation frequency were compared (Figures 9E, F). By analyzing the mutation annotation files of the TCGA-LUAD cohort, we found that there were noteworthy differences in mutation profiles between the low- and high-risk subgroups. These results may provide insight into understanding the mechanisms of LUAD ferroptosis status and gene mutations in immune checkpoints.

### LncRNA risk score and immune cell infiltration (ICI)

**FIGURE 10:**
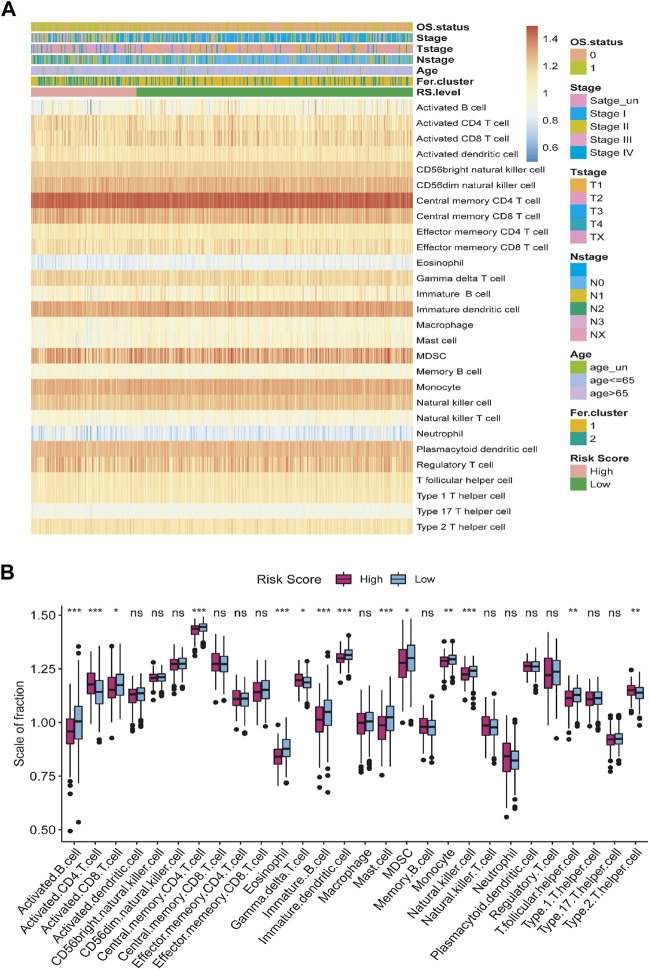
The relationship between tumor risk score and immune cells infiltration. **(A)** Heat map of the distribution of the immune cells infiltration. **(B)** Box plot of the difference in immune cells infiltration between high- and low-risk score group. *p < 0.05, **p < 0.01, ***p < 0.001, ns = no significance.

To investigate the relationship between risk score and tumor immune microenvironment, we used GSEA to assess the state of infiltration of 28 different immune cells from the TCGA-LUAD dataset (Supplementary Table S5). As a whole, LUAD patients had a high infiltration ratio of CD56^+^ dim natural killer cells, central memory CD4^+^ T cells, central memory CD8^+^ T cells, immature dendritic cells, myeloid-derived suppressor cell (MDSC), monocytes, natural killer cells, plasmacytoid dendritic cells, and regulatory T cells. LUAD tissues were less infiltrated by neutrophils, eosinophils, and type 17 T helpers (Figure 10A).

According to our hypotheses test, the infiltration level of active CD4^+^ T cells was significantly higher in the group with high-risk score than in the group with low-risk score. In contrast, the infiltration of activated B cell, activated CD8^+^ T cell, central memory CD4^+^ T cell, eosinophil, γδ-T cell, immature B cell, immature dendritic cell, mast cell, monocyte, natural killer cells, T follicular helper cells, and type 2 T helper cells in high-risk score group were significantly lower than in the low-risk score group (Figure 10B).

### The lncRNA risk score had a good predictive ability in evaluating the response of immunotherapy

**FIGURE 11:**
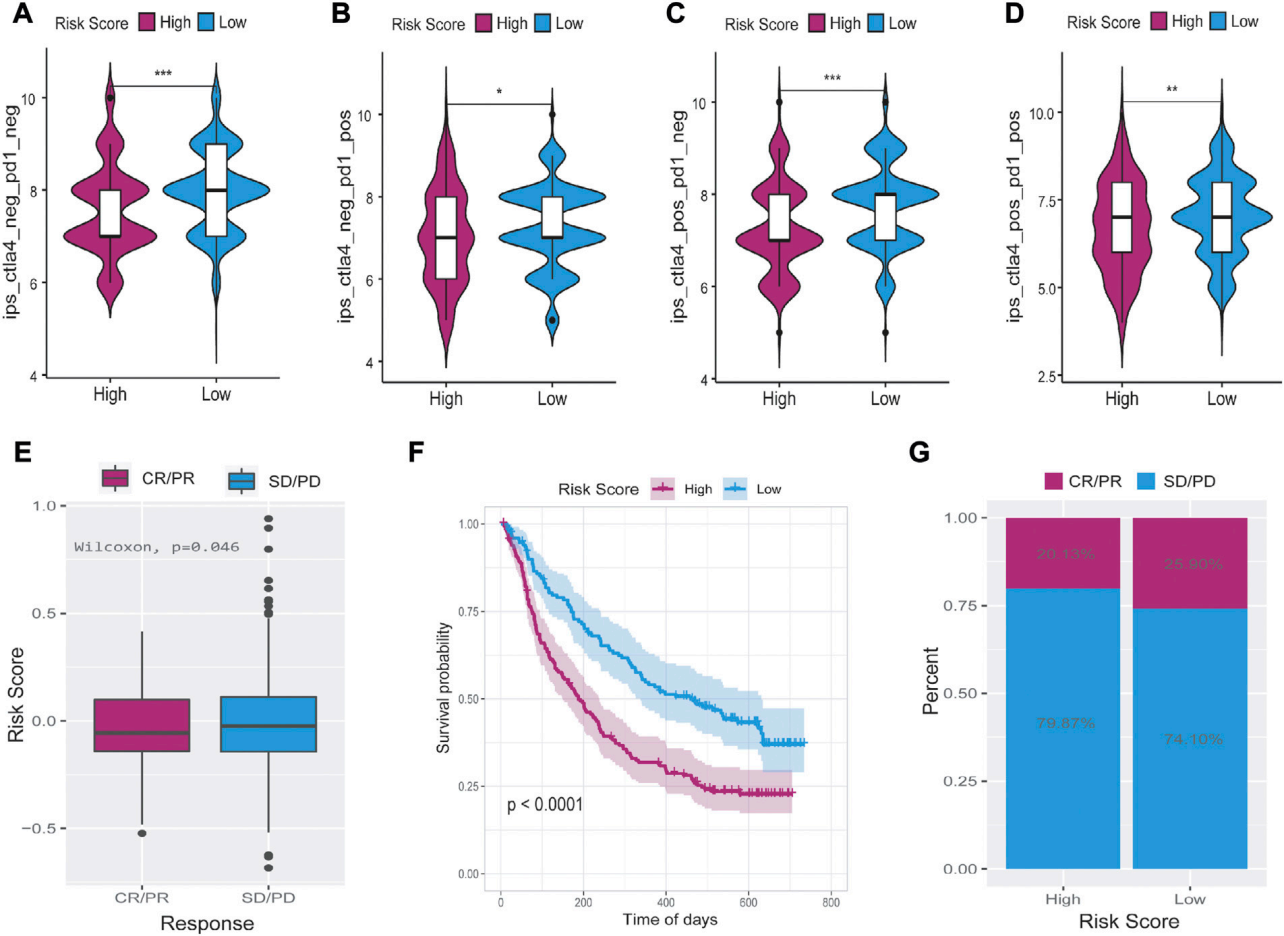
The relationship between tumor risk score and the response of immunotherapy. **(A–D)** Immunophenoscore was significantly higher in the low-risk group than in the high-risk score group. **(E–G)** Low-risk score patients who received anti-PD-L1 treatment had better responses to immunotherapy and significantly longer survival time than high-risk score patients. CR = complete response, PR = partial response, PD = progressive disease, SD = stable disease. *p < 0.05, **p < 0.01, ***p < 0.001.

To explore the predictive ability of risk score in predicting the benefit of immunotherapy, we analyzed the immunophenoscore (IPS) of samples from the TCIA database and the IMvigor210 cohort of immunotherapy patients (http://researchpub.gene.com/IMvigor210CoreBiologies). Multiple tumors can be predicted to respond to immunotherapy based on IPS, which can determine whether they are immunogenic. In Figures 11A–D, we found four types of low-risk score, namely, ips_ctla4_neg_pd1_neg, ips_ctla4_pos_pd1_neg, ips_ctla4_neg_pd1_pos, and ips_ctla4_pos_pd1_pos. IPS scores of patients in the low-risk group were significantly higher than those in the high-risk group, suggesting that immunotherapy was more likely to be effective. Patients who received anti-PD-L1 immunotherapy in the IMvigor210 cohort were divided into high- and low-risk groups. As a result, the group with low-risk scores showed a higher objective response to anti-PD-L1 therapy (Figure 11E). Moreover, patients with low-risk scores lived signifificantly longer than patients with high-risk scores (Figure 11F), and the increased risk in the IMvigor210 cohort correlated with the higher objective response rate (Figure 11G). In summary, these results suggest that the ferroptosis-related lncRNAs-based risk score may indicate the response to immunotherapy in LUAD.

## Discussion

As the most common histological type of lung cancer, LUAD accounts for 40%–50% of all lung cancer cases (Bray et al., 2018). It severely affects human health and possesses both extremely high morbidity and mortality (Cheng et al., 2021). Despite great efforts having been made in developing novel treatments, however, LUAD still received a poor prognosis (Hirsch et al., 2017). In recent years, studies have demonstrated that ferroptosis is an important regulatory mechanism for tumor growth and is important for chemoradiotherapy and immunotherapy of tumors (Chen et al., 2021). In addition, lncRNAs have been a major focus of research into ferroptosis. However, the underlying relationship between ferroptosis-associated lncRNAs and the prognosis of LUAD patients remains quite limited. In this study, the expression profiles of ferroptosis-related genes in TCGA-LUAD dataset showed individual heterogeneity. Moreover, the expression profiles were correlated with the overall survival (OS) of LUAD patients. We also found that gene mutations could affect the expression of ferroptosis-related genes. Our results were then used to construct the risk score model with 13 ferroptosis-related lncRNAs. In univariate and multivariate Cox regression analysis, the risk score model was found to be a relatively independent prognostic indicator of the clinical features of LUAD patients. In addition, this study indicated the risk score model can well evaluate the benefit of LUAD patients receiving immunotherapy.

Liu et al. established the ferroptosis potential index (FPI) to reveal the functional roles of ferroptosis and found high FPI predicted poor prognosis in several tumors, highlighting the potential value of cancer classification based on ferroptosis-related genes expression (Liu et al., 2020). As a result of the expression of tumor ferroptosis-related genes and consensus clustering, we divided the samples into Fer-1 and Fer-2 groups. It was interesting to note that patients in the Fer-1 group had a median survival time of 898 days, significantly longer than Fer-2 group patients, who had a median survival time of 685 days. The differences in survival time between the two ferroptosis subtypes were probably determined by differences in biological functions and signaling pathways as well as differences in immune cell infiltration. There seems to be a close relationship between Fer-1-enriched pathways and biological processes related to xenobiotic stimulus, hormone metabolism, and antibiotic metabolism. While Fer-2 was mostly enriched in viral entry into the host cell, leukotriene metabolism, and fluid transport. In addition, we discovered samples from Fer-1 were significantly more infiltrated with mast cells, immature B cells, eosinophils, activated B cells, activated dendritic cells, and immature dendritic cells than samples from Fer-2. In early-stage LUAD patients, mast cell abundance was associated with prolonged survival (Bao et al., 2020). Also, Han et al. found that upregulated glucose-6-phosphate isomerase (GPI) was associated with poorer survival, clinical stage, N stage, and primary therapy outcomes in LUAD. While GPI expression was negatively correlated with infiltrating levels of CD8^+^ T cells, central memory T cells, dendritic cells, macrophages, mast cells, and eosinophils (Han et al., 2021), which is consistent with our study findings. Thus, this result showed the value of the classification of Fer-1 and Fer-2 in predicting the survival of LUAD patients.

A total of 13 ferroptosis-related key lncRNAs were identified by LASSO regression. What’s more, a risk score model associated with tumor immune cell invasion was constructed based on these 13 lncRNAs. Interestingly, the risk score not only showed the ability to predict the overall survival of LUAD patients but was also associated with tumor mutation burden and evaluating the response of immunotherapy. Among the 13 key lncRNAs, LINC01352 is an important prognostic risk assessment factor for LUAD (Lu et al., 2021). By down-regulating miR-423-3p and inducing tumor suppressor protein p21, ZNF674-AS1 inhibits NSCLC growth. As a result, the low survival rate of NSCLC patients is significantly correlated with ZNF674-AS1 downregulation (Liu Y. et al., 2021). Linc00324 is over-expressed in a variety of cancer cell lines and tumoral tissues. Some researchers believe LINC00324 can be regarded as a promising candidate for the development of diagnostic and prognostic panels, what’s more, can be used as a therapeutic target for a wide range of cancers (Ghafouri-Fard et al., 2022). A study suggested that Linc00324 overexpression accelerated the proliferation, migration, and invasion of LUAD cells by activating miR-615-5p/AKT1 axis (Zhang L. et al., 2021). CRNDE is a long non-coding RNA that has been demonstrated to be involved in multiple biological processes of different cancers as well as a potential diagnostic biomarker and prognostic predictor (Lu et al., 2020). Among the downstream targets of CRNDE, miR-641, CDK6, and miR-338-3p promote lung cancer cell proliferation and inhibit cell apoptosis (Fan et al., 2019; Jing et al., 2019). There have been reports that plncRNA-1, also known as CBR3-AS1, has different effects on different kinds of tumors. As an example, CBR3-AS1 modulates JNK1/MEK4 and enhances MAPK signaling by binding miR-25-3p competitively, suggesting it is a breast cancer prognosis marker (Zhang M. et al., 2021). Further, CBR3-AS1 is a poor prognostic molecule for osteosarcomas and colorectal cancer. Accordingly, high levels of CBR3-AS1 inhibit colorectal cancer metastasis by targeting the PI3K/Akt pathway (Zhang et al., 2018). Min Hou et al. found that CBR3-AS1 is associated with the prognoses of LUAD by activating the signal from the Wnt/β-catenin. (Hou et al., 2021). Despite its antisense lncRNA gene status, little is known about the role of ADPGK-AS1 in lung cancer. However, it has been reported to contribute to cervical, gastric, and colorectal cancer (Nagasaki et al., 2012; Jiang and Wang, 2020; Zhong Q. et al., 2021). ADPGK-AS1 has been shown to inhibit miR-205-5p downregulation in pancreatic cancer, which is negatively correlated with cancer cell proliferation, migration, and invasion, and positively correlated with apoptosis rates. The EMT process can thus be strongly induced *in vivo* by it (Song et al., 2018). Liu et al. demonstrated that downregulation of OGFRP1 inhibited the progression of NSCLC through miR-4640-5p/eIF5A axis (Liu X. et al., 2021). Furthermore, it has been reported that OGFRP1 is highly expressed in NSCLC tissues and significantly correlated with the prognosis of LUAD patients (Cui et al., 2021). As another core ferroptosis-related lncRNA noted in this study, APTR has been shown to reduce miR-132-3p and enhance YAP1 expression, which in turn promotes osteosarcoma progression (Guan et al., 2019). However, no study to date had demonstrated the relationship between APTR and lung cancer. It showed that AC008278.2 was a protective lncRNA was one of 19 genomic instability-related lncRNAs that correlated with somatic mutation pattern, immune microenvironment infiltration, immunotherapeutic response, drug sensitivity, and survival of NSCLC patients (Zhang et al., 2022). While as for PAN3-AS1 and AC093911.1, little has been studied in current diseases or molecular mechanisms. Further excavation is required to understand the role of these lncRNAs in lung cancer development.

TMB has emerged as a promising novel biomarker in predicting the prognosis and immune response in cancers, although the effect and the prognostic role of the TMB on outcomes varied dramatically across cancer types (Hellmann et al., 2018; Wang Z. M. et al., 2021). There are researches showed that higher TMB tends to form more new antigens, making tumors more immunogenic, improving clinical response to immunotherapy, and prolonging the overall survival (Lv et al., 2020; Wu et al., 2020). This is consistent with that patient in the high TMB scores group has better OS in our study. However, there are also studies showing the opposite. A study by Wang et al. found high TMB had a significantly poor prognosis in thymic epithelial tumors patients (Wang Z. M. et al., 2021). While Gao et al. discovered that higher TMB had a negative correlation with the prognosis of pancreatic ductal adenocarcinoma (Gao et al., 2020). The results of this study showed risk score had a modest positive correlation with TMB score, however, the risk score was negatively correlated with patients’ OS, indicating an independent role of the risk score in predicting the response to immunotherapy in LUAD patients.

Harnessing an anti-tumor immune response has long been a fundamental strategy in cancer immunotherapy. According to the previously proposed tumor immunoediting hypothesis, tumor cells entering the immune escape phase can create an immunosuppressive state within the tumor microenvironment by subverting the same mechanisms that under normal conditions help regulate the immune response and prevent damage to healthy tissue (Carbone et al., 2015). In the last decade, higher objective response rates have been observed by targeting the PD-L1/PD-1 immune checkpoint pathway. This stems from distinct mechanisms of action that restore tumor-induced immunity deficiency selectively in a tumor microenvironment (TME) (Sanmamed and Chen, 2018). The therapeutic efficacy of these anti-PD1 therapies relies on endogenous tumor-antigen-specific T cells that are functionally held in check in the TME due to PD-L1 inhibitory signaling through PD-1. Anti-PD therapy results in the adaptive increase of functional T cells, which translates into tumor regression (Herbst et al., 2022). Until now, immunotherapy has shown considerable clinical success in the treatment response of many LUAD patients. Using T cells, monoclonal antibodies, or immune checkpoint inhibitors, immunotherapy stimulates the immune system to attack tumor cells (Forde et al., 2018; Passiglia et al., 2018). What’s more, growing studies have reported that the immune-related features of cancers such as the intensity of CD4^+^ T cells and CD8^+^ T cell infiltrates, macrophages, and natural killer (NK) cells, different B cell sub-populations were correlated with immunotherapeutic responsiveness in lung cancer (Stankovic et al., 2018). In this present study, we found a functional enrichment analysis that suggested that ferroptosis-related lncRNAs were mainly involved in immune pathways. Besides, immature dendritic cells, myeloid suppressive cells, monocytes, and regulatory T cells displayed a high level of LUAD. However, neutrophils, eosinophils, and type 17 T helper cells were the major low-level infiltrating cells. Additionally, our results revealed the relationship between immune cell infiltration (ICI) and the survival of LUAD patients. Based on these findings, these ferroptosis-related lncRNAs provide potential targets for combined treatments with immune checkpoint inhibitors.

There are some limitations of our study. Firstly, only data obtained from TCGA was used to construct a ferroptosis-related lncRNA prognostic model and to evaluate its validity. Secondly, the number of lung samples used on detecting the expression levels of the identified 13 key ferroptosis-associated lncRNAs was limited. Therefore, more work is needed to fully elucidate the mechanisms underlying the effects of ferroptosis-related lncRNAs on LUAD.

## Conclusion

In conclusion, our study identified two ferroptosis subtypes to predict clinical outcomes and therapeutic responses in LUAD patients. The construction of a new risk score model with 13 ferroptosis-associated lncRNAs provides a candidate model for the evaluation of the LUAD prognosis. Our results demonstrate that LUAD patients in the high-risk score group presented worse OS, higher TMB, and lower immune activity. This study might contribute to the optimization of risk stratification for survival and personalized management of LUAD patients.

## Data Availability

The datasets presented in this study can be found in online repositories. The names of the repository/repositories and accession number (s) can be found in the article/Supplementary Material.

## Supplementary material

SUPPLEMENTARY FIGURE S1 Waterfall chart of gene mutation in TCGA-LUAD dataset.

SUPPLEMENTARY FIGURE S2 The different expressions of 60 ferroptosis-related genes between TP53 mutation and wildtype in the TCGA-LUAD dataset.

SUPPLEMENTARY FIGURE S3 The different expressions of 60 ferroptosis-related genes between TTN mutation and wildtype in the TCGA-LUAD dataset.

SUPPLEMENTARY FIGURE S4 The correlation between the 60 ferroptosis-related genes in the TCGA-LUAD dataset.

SUPPLEMENTARY TABLE S1 Clinical information statistics of TCGA-LUAD data set.

SUPPLEMENTARY TABLE S2 Differentially expressed genes between the ferroptosis subtypes.

SUPPLEMENTARY TABLE S3 LncRNAs are co-expressed with ferroptosis-related genes.

SUPPLEMENTARY TABLE S4 Thirty-nine lncRNAs retained in the training set after the screening.

SUPPLEMENTARY TABLE S5 Infiltration state of 28 types of immune cells in the TCGA-LUAD dataset.

SUPPLEMENTARY TABLE S6 The primer sequences of the tested lncRNAs.
