# Supplementary figures and images for: Prognostic markers of ferroptosis-related long non-coding RNA in lung adenocarcinomas
Source: Front Genet. 2023 Feb 27;14:1118273. doi: 10.3389/fgene.2023.1118273 (PMC10009162; doi:10.3389/fgene.2023.1118273)

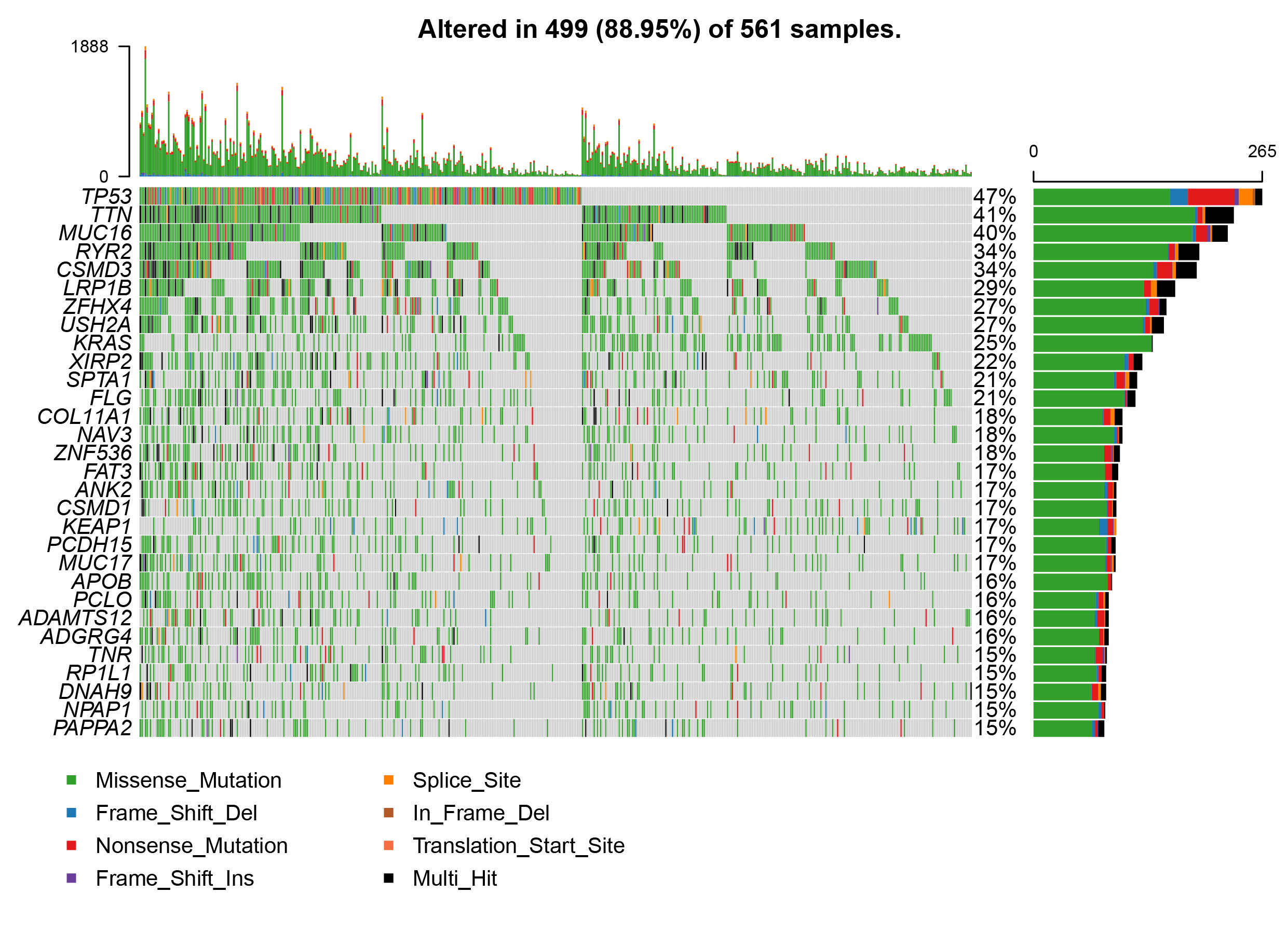

Supplement: Supplementary file 4 [file Image1.JPEG]

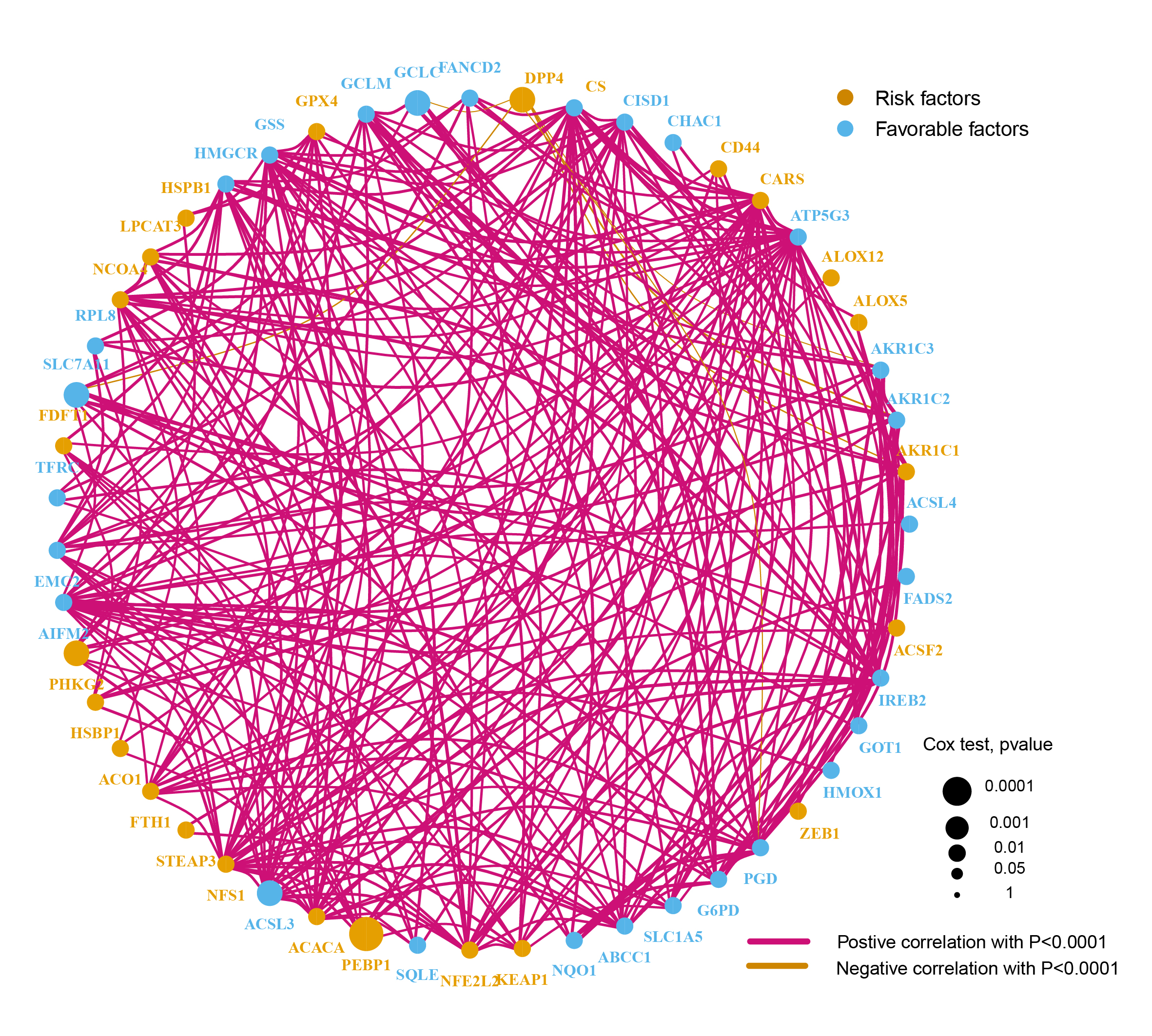

Supplement: Supplementary file 5 [file Image4.JPEG]
